# A Cross-Sectional Analysis of Students’ Intuitions When Interpreting CIs

**DOI:** 10.3389/fpsyg.2018.00112

**Published:** 2018-02-16

**Authors:** Pav Kalinowski, Jerry Lai, Geoff Cumming

**Affiliations:** ^1^School of Psychological Science, La Trobe University, Bundoora, VIC, Australia; ^2^Cogstate, Melbourne, VIC, Australia

**Keywords:** confidence intervals, misconceptions, teaching, statistical intuitions, subjective likelihood distribution

## Abstract

We explored how students interpret the relative likelihood of capturing a population parameter at various points of a CI in two studies. First, an online survey of 101 students found that students’ beliefs about the probability curve within a CI take a variety of shapes, and that in fixed choice tasks, 39% CI [30, 48] of students’ responses deviated from true distributions. For open ended tasks, this proportion rose to 85%, 95% CI [76, 90]. We interpret this as evidence that, for many students, intuitions about CIs distributions are ill-formed, and their responses are highly susceptible to question format. Many students also falsely believed that there is substantial change in likelihood at the upper and lower limits of the CI, resembling a cliff effect (Rosenthal and Gaito, 1963; Nelson et al., 1986). In a follow-up study, a subset of 24 post-graduate students participated in a 45-min semi-structured interview discussing the students’ responses to the survey. Analysis of interview transcripts identified several competing intuitions about CIs, and several new CI misconceptions. During the interview, we also introduced an interactive teaching program displaying a *cat’s eye* CI, that is, a CI that uses normal distributions to depict the correct likelihood distribution. Cat’s eye CIs were designed to help students understand likelihood distributions and the relationship between interval length, *C*% level and sample size. Observed changes in students’ intuitions following this teaching program suggest that a brief intervention using cat’s eyes can reduce CI misconceptions and increase accurate CI intuitions.

## Introduction

**FIGURE 1:**
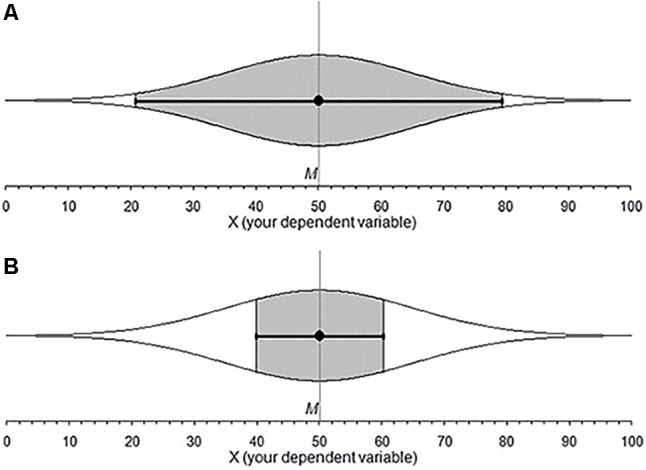
Cat’s eye confidence intervals, points inside a CI are not equally likely to land on the μ. **(A)** 95% CI and shows 95% of the overall distribution. **(B)** A 50% CI for the same data: The gray area shows that much less of the overall distribution is accounted for.

Think of a 95% confidence interval (CI). Do all points inside a CI have the same likelihood of capturing the population parameter? Are some points in the interval more likely than others? The former intuition implies a uniform distribution, the latter may describe a normal or *t* distribution. We investigated intuitions about the probability distribution behind a CI. We label these cognitive representations of CI distributions *subjective likelihood distributions* (SLDs). The study of SLDs may provide insight into the interactive nature of various CI misconceptions. If so, it may be possible to eliminate a suite of misconceptions by simply correcting a person’s SLD, perhaps by teaching CIs with more informative and intuitive graphics. An example is the cat’s eye graphic (**Figure 1**), where an ordinary CI is overlaid with a normal distribution (Cumming, 2007, 2012). In this paper, we explore graduate student intuitions about CIs and suggest a tool to improve those intuitions.

### The Cognition of Confidence Intervals

Using CIs instead of *p* values has been advocated by many proponents of statistical reform in psychology (Schmidt, 1996; Fidler and Loftus, 2009; Cumming, 2012). Some researchers have suggested that CIs are easier to interpret than traditional NHST (Schmidt and Hunter, 1997). Such claims have been persuasive. For example, since 2014 effect sizes and CIs have been required by the high profile journal *Psychological Science* to “avoid problems associated with null-hypothesis significance testing^1^.” These arguments and interventions have not been without controversy, with others arguing that CIs are also poorly understood, or philosophically flawed (Morey et al., 2015). For example, Hoekstra et al. (2014) demonstrated that both researchers and students endorsed on average three misinterpretations of CIs when presented with six incorrect statements interpreting CIs. Experience with statistics seemed to have no correlation with the number of false interpretation endorsed. The authors concluded that many students and researchers generally do not understand CIs. Miller and Ulrich (2016) argued that the statements presented by Hoekstra et al. (2014) were misleading, and could be interpreted in a correct way. Morey et al. (2016) responded by arguing that there was no evidence that the participants interpreted the questions using the alternative interpretations. Moreover, although correct, the alternative interpretations fail to indicate the location of the parameter, or the reference class of the population. Garcia-Perez and Alcala-Quintana (2016) replicated and extended Hoekstra et al.’s (2014) research by adding two correct statements. Participants endorsed correct and incorrect statements equally. The current research contributes to these ongoing questions about CI interpretation.

### Interpreting a CI

A CI is a point estimate surrounded by uncertainty. When sampling from a population distribution our sample mean gives us an estimate of the population mean (μ). As sampling is repeated, and new sample means are calculated the sample means vary. As sampling continues (drawing samples of the same *n*) the sample means tend to bunch causing a mean heap (central tendency of repeated sampling). The standard deviation of this mean heap is the standard error. Approximately two of these standard errors either side of the population mean will capture 95% of the sample means with 2.5% of sample means falling outside the upper limit and 2.5% falling outside the lower limit. If we place the same error bars on a sample mean we have a 95% CI. In an infinite number of repeated trials 95% of these CIs will capture μ. The confidence level (*C%* level) is the percentage of the repeated sample CIs that will capture μ (or other chosen population parameter). Keep in mind that it is the CI that varies, while μ is fixed but unknown.

The relative likelihood of each point across an interval falling on μ is not equal (Cumming, 2007). Rather, points closer to the sample mean are more likely to fall on the population parameter than those further away; the relative likelihood of points across and beyond a CI (given σ) is distributed normally; (**Figure 1**). If σ is not known, the relative likelihood follows a *t* distribution. The *C%* level is the percentage of the overall distribution that a CI includes. CIs with different *C%* levels indicate different percentages of the likelihood distribution, which remains the same. The likelihood profile inside a CI is very different for a 50% CI and a 95% CI. Looking at the gray area in **Figure 1** we can see that a 50% CI has a substantially different shape to a 95% CI. The 95% CI has a clear normal distribution, while the 50% CI shows gradual change because it is capturing only the middle portion of the overall distribution.

### Subjective Likelihood Distributions

We use the term SLD to refer to a cognitive representation of the relative likelihood of each point across and beyond a CI in landing on the population parameter. For example, a uniform SLD reflects the (incorrect) belief that every point inside a CI is equally likely to have landed on μ.

We can think of a CI as comprised of three main sections: (1) The interval between the lower limit and upper limit, (2) The limits themselves, and (3) The regions beyond the limits. Observing a person’s judgments about likelihood of points in these sections allows us to plot a shape that represents their SLD. For example, if a person judges all the points in Section 1 as equally likely, the points in Section 3 as substantially less likely than those in Section 1 and as all equally unlikely, then their cognitive representation of a CI can be plotted as a square, or uniform distribution. Note that this may not necessarily mean that if asked explicitly ‘what distribution does a CI have?’ they would answer ‘uniform.’ In fact, we suspect that many students could not answer this question at all. What we elicit in our judgment tasks of the likelihood of points in different sections is their intuition—a hint at what their SLD might be.

One important determinant of the shape of an SLD may be whether an individual thinks of a CI as a substitute for Null Hypothesis Significance Tests (NHSTs). Poitevineau and Lecoutre (2001) asked researchers to rate their confidence that an effect existed at 12 different *p*-values ranging from 0.9 to 0.001 (**Figure 4**). They identified three categories of response: An all-or-none curve (very high degree of confidence when *p* < 0.05 and almost no confidence otherwise), a negative linear curve and a decreasing exponential. The second and third categories may reflect SLDs that follow Fisher’s significance testing model. Fisher saw *p*-values as indicators of strength of evidence, as the smaller the *p*-value, the stronger the evidence against the null hypothesis (Fisher, 1956/1990). The all-or-none category suggests Neyman–Pearson dichotomous decision making. If people think about CIs as substitutes for *p*-values, we would expect at least those three shapes when categorizing our students’ SLDs. There is independent evidence that researchers do interpret CIs as substitutes for *p-*values: In an analysis of 145 text responses on a CI judgment task, Coulson et al. (2010) found that 44% of researchers in psychology, behavioral neuroscience, and medicine invoked NHST when interpreting CIs.

Strict adherence to the Neyman–Pearson decision making model may lead to uniform SLDs with sharp cliffs at the lower and upper limits (or perhaps the inability to make any statement about relative likelihood when looking at a single CI), whereas Fisherian sympathies may lead to SLDs that reflect a more gradually decreasing distribution such a normal or *t-*distribution. Hybrid logic (a combination of both Fisherian hypothesis testing and Neyman–Pearson decision making as defined by Gigerenzer, 1993) may produce a hybrid shape—for example a flat-topped distribution with a large drop in likelihood at the upper and lower limits reflecting Neyman–Pearsons belief that no statement of likelihood can be made from a single confidence interval followed by gradually sloping sides outside the CI reflecting a Fisherian belief that statements of relative probability can be made from single *p*-values.

## Experiment 1: Interactive Student Survey

### Introduction to Tasks

Our research program followed previous experiments (Cumming et al., 2004; Fidler and Loftus, 2009) in exploring student and researcher intuitions and misconceptions when interpreting CIs. There has been a long and persistent call to ban NHST and CIs have been identified as a possible tool to help reduce NHST misconceptions. We believe that when used as a form of effect size estimation rather than hypothesis testing, CIs provide a simple and intuitive statistical tool if understood. However, we do not yet know how students approach CIs when reading and interpreting empirical literature.

#### Tasks 1, 2, and 3: Student Intuitions on Likelihood Distributions

Experiment 1 aimed to elicit students’ SLDs for CIs. Although this study was exploratory, we hypothesized that shape categories would include: (1) a square shape or uniform distribution, (2) a linear decrease or triangle distribution, (3) a normal distribution, and (4) various hybrids of the previous three. We used three different tasks to elicit the students’ distributions, and assessed consistency between tasks.

#### Task 4: The Relationship between Width and *C%* Level

Fidler and Loftus (2009) found that 73% of first year undergraduates believed a higher *C*% level would have a shorter interval for the same data. Experiments on interval judgment provide insights that may inform our understanding of this misconception. Task 4 consisted of an interval adjustment task. This was to explore the prevalence of this misconception in students with more statistical experience.

### Method

#### Participants

Final year honors undergraduates and graduate students were recruited through official course online noticeboards, email, social networking websites, and word of mouth, 101 students agreed to participate. Two thirds (66%) of students identified psychology as their main discipline; other disciplines include social science (13%), neuroscience (6%), and medicine (5%). The remainder (10%) did not identify their discipline. Most students (63%) were enrolled in a post graduate program, and the remaining students where completing their honors (fourth year undergraduate).

#### Materials

We developed an internet survey consisting of three judgment tasks. The first task in Experiment 1 asked students to rate the relative likelihood of points across a CI landing on μ, the parameter being estimated. The second task asked students to choose the shape that best represented their intuitions about the distribution of a CI from a set of six multiple choice options.

Task 3 presented students with a 50% CI, and asked them to adjust its width to correspond to a 80% CI, and a 95% CI. This task was repeated for adjusting a 95% CI to an 80% and a 50% CI. We also asked two open ended questions about how the student approached the tasks, and about their familiarity with the concept of likelihood distributions. Finally we asked for the students’ familiarity with CIs and some demographic details.

#### Procedure

Potential student respondents were emailed an invitation with a link to the survey, and a brief explanation of the tasks involved in the questionnaire. We also invited potential respondents to pass on the link to others who might be interested in participating. The survey was designed to take around 15 min to complete, however, no time limit was set. The average time taken to complete the survey was 16 min.

##### Task 1: Judging the likelihood of points inside and outside a CI, relative to the sample mean

**FIGURE 2:**
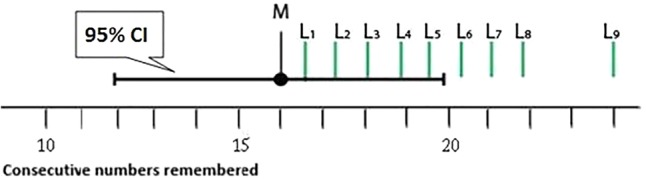
A CI was presented as part of Task 1. Students rated the likelihood of points L_1_ to L_9_ relative to the sample mean (*M*) on a 19-point scale.

Students were provided with a simple research vignette as well as **Figure 2**. They were asked to rate the likelihood of points L_1_ to L_9_, each relative to the sample mean (*M*) on a 19-point scale.

The 19-point scale ranged from (1) ‘More likely [to] land on the μ,’ (3) ‘About equally likely [to] land on the μ,’ (5) ‘Very slightly less likely to be the μ’ to (19) ‘Almost zero likelihood.’ The range was skewed so that students had 16 choices below ‘About equally likely…’ An identical figure (except for the label ‘50% CI’) and a comparable vignette were presented, for a 50% CI.

**FIGURE 3:**
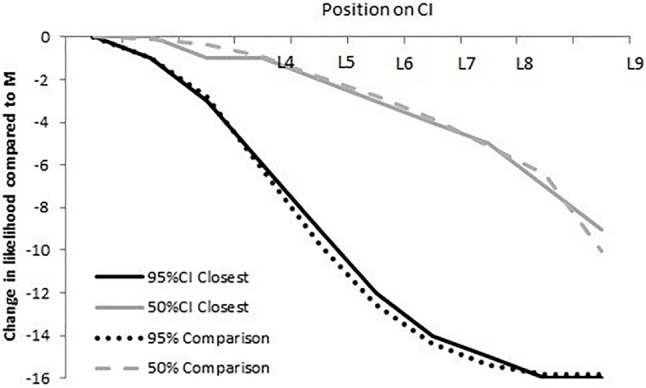
The upper dashed and lower dotted lines represent the differences in relative likelihood from *M* to L*i* (*i* = 1–9) on a 16-point scale for a 50% CI (upper dashed line) and a 95% CI (bottom dotted line). The solid curves illustrate the closest possible fit (with *R*^2^ = 0.99) between a given response and its normative distribution (dotted or dashed curves), using the response scale used in our survey.

*Classification of student responses*. Correct: We used a comparison distribution (**Figure 3**) to identify students with a normally distributed SLD. The correct distribution was determined by plotting the relative likelihood for a 50% CI and a 95% CI using a 16-point scale—the portion of the original 19-point scale that ranged from equally likely to almost zero likelihood.

**FIGURE 4:**
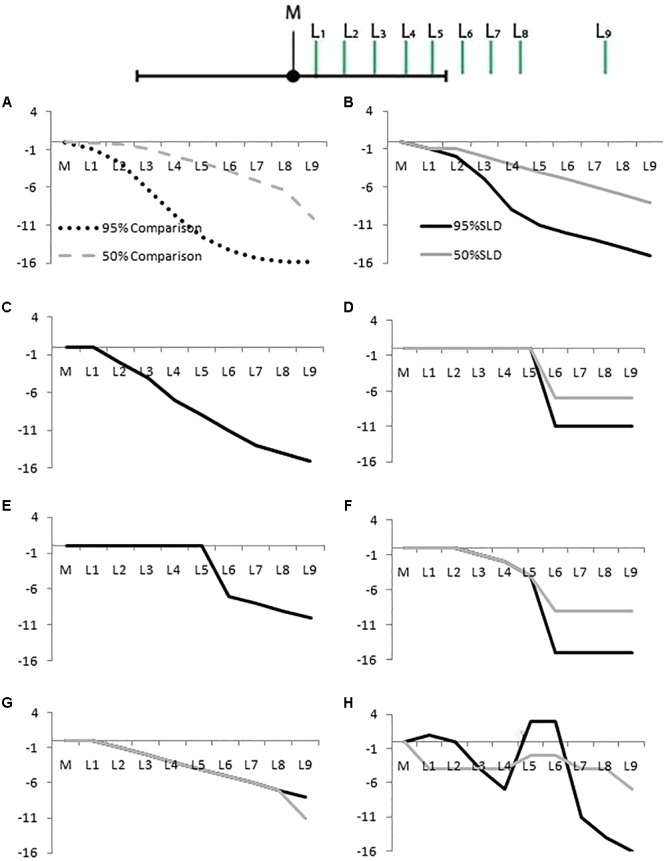
Likelihood distribution curves, for 50% (gray lines) and 95% CIs (dark lines). **(A)** The correct shape for a 95% CI (dotted line) and 50% CI (dashed line). The other panels show examples of student responses classified as: **(B)** Correct, **(C)** Gradual Curve (gray and dark lines coincide), **(D)** Square, **(E)** Mesa (gray and dark lines coincide), **(F)** Half Circle, **(G)** Triangle, and **(H)** Other. The *X*-axis represents points L_1_ to L_9_ as in **Figure 1**. The *Y*-axis represents the difference in rated likelihood on a 19-point scale.

A student’s SLD was categorized as Correct if the comparison distribution accounted for at least 97% of variance of the students’ SLD. It was sometimes difficult to demarcate this category from the Gradual Curve and the Triangle categories below. Even though we set the *R*^2^ cut off very high (97%) we acknowledge that there is still overlap between these categories, as **Table 1** shows.

**Table 1:** Percentage [and 95% CIs] of students with each shape in Task 2.

Shape	% Students (95% *C* level)	% Students (50% *C* level)	% Students (consistent across 95% and 50% *C* levels)	^b^
Correct	15 [9, 23]	17 [11, 25]	10 [6, 17]
Bell shape	12 [7, 20]	18 [11, 26]	5 [2, 11]
Triangle	4 [2, 10]	7^a^ [3, 14]	2^a^ [0, 7]
Half Circle	10 [6, 17]	5 [2, 11]	4 [2, 10]
Mesa	16 [10, 24]	12 [7, 17]	9 [5, 16]
Square	19 [12, 28]	13 [8, 21]	11 [6, 18]
Other	25 [17, 34]	36 [27, 45]	14 [8, 22]

*The first two columns of percentages show percentages of each SLD relating to a 95% CI and a 50% CI, respectively; the third column of percentages shows the percentage of students who consistently held the same shape SLD over both *C*% levels.*

*^*a*^Because a Triangle shape has a high *R*^*2*^ with the correct category for 50% CIs these students could be categorized as both ‘Triangle’ and ‘Correct’ and have been classified twice.*

*^*b*^Overall 46% of participants had inconsistent SLDs.*

Gradual Curve: We categorized students’ SLDs as Gradual Curve for both 50% and 95% CIs if the curve had a curvilinear drop across the CI. This category was very lenient. If an SLD was not classified as Correct, had no cliff between points L_5_ and L_6_ (these points lie either side of the upper limit of the CI, see **Figure 2**), and did not have a linear drop (see Triangle category below), it was classified as a Gradual Curve. The Gradual Curve category was not as strict as the correct category but we still regard it as a fairly good student intuition.

Square and Mesa: For Square or Mesa classifications, a student must have rated L_1_ to L_5_ as equally likely, and then dropped the likelihood rating between points L_5_ and L_6_ A Square is defined by final points (L_6_ to L_9_) with equal likelihood ratings whereas a Mesa shows a continued reduction in likelihood after from L_6_ to L_9_.

Half Circle: For the Half Circle classification several points from (L_1_ to L_5_) had to show a drop in likelihood. However, the drop in likelihood between L_5_ and L_6_, located just inside and just outside the CI, had to be greater than for the other pairs of Ls.

Triangle: A SLD was classified as Triangle if it had a negative linear trend, that is, equal drops in likelihood over at least seven consequent points.

Other: A classification of Other was given to all SLDs that did not fit any of the above criteria, including showing no change in likelihood across any of the points (two respondents showed this response).

##### Task 2: Multiple choice task

Task 2 asked students to choose a shape that best represented their SLD from a set of six shapes (**Figure 6**). The shapes selected for Task 2 were prompted partly by considering Neyman–Pearson, Fisherian and hybrid approaches to hypothesis testing, and the patterns found by Poitevineau and Lecoutre (2001). In a sense, responses to Task 2 present a lower bound for the prevalence of each SLD. Similarly, we might think of responses to Task 1 as offering the associated upper bound on prevalence.

##### Task 3: The relationship between width and C% level

**FIGURE 5:**
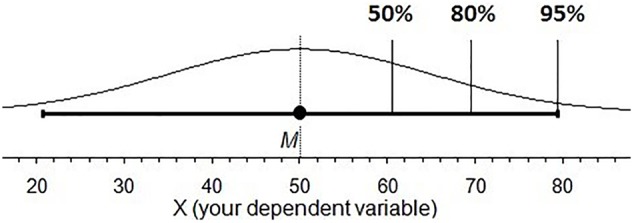
The likelihood distribution of each point of a 95% CI landing on μ. The 50, 80, and 95% marks indicate the upper limit of the CI with each of those *C%* levels.

The underlying distribution of a CI is directly linked to the width of a CI given a *C%*. **Figure 5** shows the upper limit of a CI having a *C%* level of 50, 80, and 95. Because the distribution flattens as we move further from *M*, the distance between 50 and 80 is slightly shorter than the distance between 80 and 95 even though it corresponds to twice the area under the curve.

In Task 3, students were shown a CI and asked to click on the slider to the right of the screen to set the lower interval to what they felt was the right length at a different *C%* level, 50 and 80 when given a 95% CI (Question 1), and 80 and 95 when given a 50% CI (Question 2).

### Results

#### Task 1: Judging the Likelihood of Points Inside and Outside a CI, Relative to the Sample Mean

Percentages of students for all shape categories for Task 1 are presented in **Table 1**.

#### Task 2: Multiple Choice Task

**FIGURE 6:**
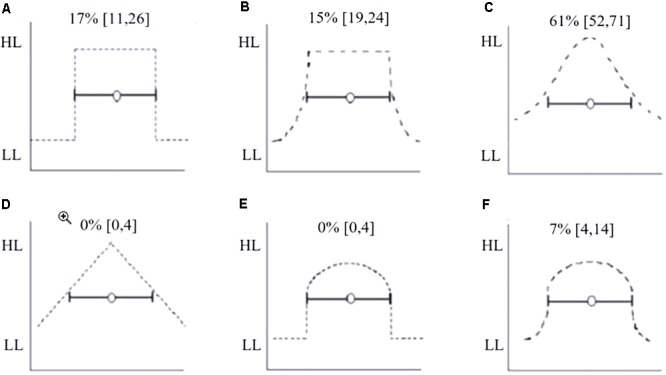
Six likelihood distributions presented as response options for Task 3. **(A)** Square, **(B)** Mesa, **(C)** Bell curve, **(D)** Triangle, **(E)** Half Circle with no change in likelihood after the lower limit, **(F)** Half Circle with a gradual change in likelihood after the lower limit. Students selected which distribution best matched their own SLD of a 95% CI. The percentage of students selecting each option is shown directly above the distribution. The *Y*-axis represents likelihood ranging from high likelihood (HL) to low likelihood (LL).

**Figure 6** shows the percentage of students who chose each of the likelihood distributions shown as the best representation for their SLD. One third of students (31%, 31 of 101, [24, 43]) selected Square (A) or Mesa (B) shapes as the best representations of their SLD. We interpret these responses as evidence of a uniform SLD within the CI.

#### Task 3: The Relationship between Width and *C%* Level

Three quarters (75%, [67, 84]) of students adjusted the intervals in the correct direction. However, adjustments were on average not sufficiently large. When asked to adjust from an initial *C%* level of 95, the average estimated width of a 50 and 80% corresponded to a 63% CI, and 86% CI, respectively. In other words, the intervals were left too long. When asked to adjust from initial *C%* level of 50, students’ estimated widths of an 80% CI and 95% CI corresponded to 79% and 92%, respectively. In this case, the intervals were left slightly too short. Also, 30% of the responses indicated that a 50% CI is exactly half the length of a 95% CI, when in fact a 50% CI is approximately one third of the width of a 95% CI.

A quarter of students (25%, 25 of 101, 95% CI [16, 33]), incorrectly believed that CI width would *decrease* as the *C*% level *increased*, that is, they created 95% CIs that were shorter than 80% CIs, and 80% CIs that were shorter than 50% CIs.

#### Analysis of Open-Ended Questions

We asked students two open ended questions; the first was “How did you go about answering these questions, for example, did you have any particular model in mind? Did you use any rules of thumb?” We received informative responses from 68 (of 101) of students (the remainder merely said, for example, “no” or “from lectures”).

Of the informative responses, 38% (26 of 68) mentioned having a normal distribution model in mind, for example “I believe that plausible values of a 95% CI are normally distributed around the mean. Thus, the further away a data point is from the mean in a 95% CI, the less likely it is to be the μ. Correspondingly, a 50% CI would be measuring a narrower distribution than a 95% CI.” Eight responses mentioned SD and one response mentioned SE.

Seventeen responses defined the CIs as a range. Of these, eight students mentioned that they thought everything inside the CI is equally likely. Six students mentioned that they found the tasks challenging or confusing. For example:

“There is a 95% chance that the true μ is within those parameters. I did not think the likelihood would be affected by a value’s distance to the mean as long as the values were within the 95% CI. However, when I saw the graphs, it does make more sense for the [normal distribution]…now I’m just confusing myself.”

The second open ended question was “…we introduced likelihood distributions, are you familiar with this concept? If so where have you come across it before?” We received 87% (87 of 101) usable responses. Of these, 53% expressed familiarity with likelihood distributions.

#### CI Misconceptions

Our investigation has revealed four new CI misconceptions (**Table 2**). We also observed CI misconceptions that had been identified in previous studies.

**Table 2:** Misconceptions observed in Experiment 1.

	Description of misconception
New	All points inside a CI are equally likely to land on the μ
New	All points outside a CI are equally unlikely to land on the μ
Lai et al., 2012	There is a likelihood cliff at the end of a CI (both 50% and 95% CIs)
New	50% CIs and 95% CIs have the same distribution.
Lai et al., 2012	Likelihood decreases in a linear way as we move away from the sample mean.
Fidler and Loftus, 2009	As confidence level increases, CI width decreases (for the same data).
New	A 95% CI is roughly double the width of a 50% CI.

#### Familiarity with CIs

Students were asked to give a rating of their familiarity with CIs on a six-point Likert scale that ranged from 1 “not at all familiar with CIs” to 6 “Very familiar with CIs…Often use them in research.” The median score was 4 which correspond to the statement “I have seen CIs in research and know what they tell me.” We compared the familiarity ratings of students grouped by their chosen SLDs in Task 3. The CIs overlapped considerably for all groups except Mesa. With a difference between Mesa (3.2) and Square (4.0) of 0.8 [1.4, 0.17], Mesa and Bell shaped (3.8) of 0.6 [1.0, 0.17] and Mesa and Half circle (4.1) of 0.9 [1.7, 0.19].

### Discussion

Our results indicate two main findings. First, almost three quarters (74%) of students gave a response inconsistent with the normative in at least one of the three tasks. Second, although 75% of students adjust CIs in the correct direction, one quarter (25%) of students believed that, for given data, as the *C%* level increases the width of a CI is reduced.

#### Tasks 1 and 2

There was a large difference between the proportion of ‘correct’ SLDs we elicited in Task 1 compared to the proportion who selected the correct option in the fixed choice Task 2. In Task 1, only 27% of students’ SLDs were categorized as Bell or Triangle categories which we considered the most correct answers, whereas in Task 3, 61% chose Bell. Clearly, students’ responses were heavily shaped by the question format. This perhaps reflects the fragility of their understanding of the concept of the CI distribution.

Students’ shapes suggested several different types of SLDs. The Square shape indicated dichotomous decision making, a binary decision about the importance of results based on the arbitrary limit of the CI. This kind of intuition is consistent the Neyman–Pearson dichotomous decision making in so much as it prohibits discussion of varying likelihoods within the interval. The Half circle and Mesa shapes may also reflect a tendency to interpret CIs dichotomously, however, at the same time they reveal another SLD, a gradual reduction of likelihood for values further away from the sample mean. They may thus represent a combination of Neyman–Pearson and Fisherian NHST, possibly the hybrid approach outlined by Gigerenzer (1993).

Only four students were classified as Triangle in Task 1 and nobody selected the Triangle in Task 3. It remains ambiguous whether it is just a poor approximation to a normal SLD or whether it exists as a linear SLD. If a linear SLD does exist at the very least it is not a representation of dichotomous decision making. However, we can’t rule out the former as it would be difficult to plot out an accurate Bell shape in SLD Tasks 1 and 2. This was explored further in Experiment 2.

Around half the students had the same shape for both *C*% levels. If students correctly believed that a 50% CI is a proportion of a 95% CI they would have had different shapes for the two *C*% levels (**Figure 1**). The results don’t allow us to distinguish whether students with different shapes tried to plot the different SLDs for 50% and 95% CIs, but were unsuccessful, without asking the students directly. However, we know that students who plotted the same shape for Square, Mesa, and Half Circle ignored or did not consider the logical inconsistency of identical SLDs for 50% and 95% CIs. Students in this category may not realize that a 50% CI is a portion of a 95% CI.

#### Task 3

A quarter (25%) of our student sample thought that as *C*% level increases the length of a CI decreases, contrasting with Fidler and Loftus’ (2009) findings in which 73% of first year students has this misconception. It is encouraging that the percentage of the more experienced fourth year and postgraduate students was not as high as in the Fidler and Loftus (2009) sample. At the same time, this is still a high percentage.

The ambiguity of the word ‘confidence’ may be the best explanation for the results found in both studies. Confidence is associated with surety, and more precise predictions are judged as preferable (Yaniv and Foster, 1997) to wide intervals. In the everyday usage of ‘confidence,’ precise estimates are often equated with high confidence. If a person is asked the time and they give a precise estimate “it’s between 10 and 15 min past 5 pm” they are often judged as more confident than someone with a less precise estimate “it’s between 4 pm and 6 pm.” Yet in formal statistics, for a given fixed data set, the opposite relationship exists between precision and confidence. This everyday relationship is in competition with the formal relationship, and this may have affected student responses.

#### Limitations

There are considerable differences in student judgments depending on question format. For example, in Task 2, the majority of the students (61%) identified the normal distribution as their 95% CI SLD. Yet in Task 1, only 27% student responses fit the normal distribution (Bell) category. One explanation is the availability heuristic (Tversky and Kahneman, 1971)—in Task 3, which presented multiple options, students may simply have picked the familiar normal distribution, regardless of the question at hand. However, we suspect that the availability heuristic might have only wielded such force because students’ understanding of the relevant concepts is fragile.

After accounting for limitations, there is sufficient support to believe that the SLDs elicited in Tasks 1 and 2 represent something real. This evidence comes from triangulating the elicited SLDs with open ended comments. For example, Student #8’s answers to all tasks were classified as Square and that student stated “I answered these questions believing that for a 95% CI there is a 95% chance that the true μ is within [the limits], I did not think the likelihood would be affected by a value’s distance to the mean as long as the values were within the 95% CI.” In addition only three open ended responses directly mentioned conceptual difficulty with the tasks.

It is important to consider whether these results are due to students guessing their answers given the question format or whether there are consistent and coherent beliefs. The validity of capturing arguably ‘fuzzy’ intuitions using the exactness of a 19-point scale needs to be considered. The informativeness of these qualitative responses prompted us to design a second study to interview the students and explore whether their thoughts and intuitions can be adequately measured using such tasks.

## Experiment 2

In our second study, we wanted to further explore student intuitions as well as trial a new visual CI presentation, namely, cat’s eyes. We were interested in how students would interpret the figure and to what extent it might mitigate misconceptions. Cat’s eyes were designed to provide information about the relative distribution of likelihood across a CI (Cumming, 2007, 2012) as well as the relationship between length, *C*% level, and sample size. As such, they directly confront many misconceptions mentioned in Experiment 1.

### Method

#### Participants

Of the original sample of 101 students in Experiment 1, 24 agreed to an interview. All interviewees (*N* = 24) had completed a fourth year in a psychology degree; 22 were currently enrolled postgraduate students. Interviews were conducted approximately 8–12 months after the students had completed the tasks in Experiment 1. **Table 3** shows a summary of each participant’s results in Experiment 1 Task 2.

**Table 3:** Summary of Study 2 participants’ results from Study 1 (Tasks 1 and 3).

#	SLD 95% Task 1	SLD 50% Task 1	Inverse length^a^	Double *C*% double length^b^	*C*% level of length of 50% CI Given 95% CI	*C*% level of length of 95% CI Given 50% CI
1	Bell	Bell	No	No	61.6	82.3
2	Other	Other	Yes	No	100^c^	12
3	Half Circle	Square	No	No	61.6	90.1
4	Correct	Mesa	Yes	No	100^c^	6
5	Square	Square	Yes	No	100^c^	29.2
6	Correct	Correct	No	No	41.4	93
7	Mesa	Mesa	Yes	No	100^c^	11.9
8	Other	Half Circle	No	No	42	86.6
9	Mesa	Flat line	No	No	61.6	92
10	Other	Other	No	No	55.4	93
11	Other	Bell	Yes	No	100^c^	23.6
12	Mesa	Mesa	Yes	No	100^c^	6
13	Other	Other	No	Yes	67.3	86.6
14	Correct	Correct	Yes	No	100^c^	6
15	Square	Square	No	No	70.8	89.4
16	Correct	Bell	No	Yes	67.4	92.8
17	Half Circle	Other	No	No	48.8	99.0
18	Correct	Mesa	Yes	No	100^c^	15
19	Mesa	Mesa	No	Yes	67.4	82.3
20	Bell	Bell	Yes	No	100^c^	25.9
21	Half Circle	Half Circle	No	No	55.6	96.6
22	Square	Square	Yes	No	100^c^	20.6
23	Bell	Bell	No	No	48.8	96.7
24	Square	Flat line	No	Yes	67.4	89.4

*^*a*^The inverse length misconception (a 95% CI is shorter than a 50% CI).*

*^*b*^Double *C*% level double length misconception (a shift between a 50 and 95% *C*% level doubles the length of the interval and a shift between 95 and 50% CI roughly halves the length of a 95% CI).*

*^*c*^Although theoretically a 100% CI captures all possible values the *C*% level for these intervals is so high that they are practically 100%.*

#### Materials

Students were provided with paper, and pencils, so they could annotate and add diagrams to their explanations if they chose. As the interview progressed they were gradually given copies of their answers to the original survey questions. All interviews were recorded, transcribed and coded. All transcripts were double coded. Students were also provided with a computer with the cat’s eye program developed in ESCI (Cumming, 2001–2011) during part three of the interview.

#### Procedure

The interview consisted of three parts, the first focused on Task 1 from Experiment 1, where the participants judged the relative likelihood of nine points on a CI relative to the mean. The second part explored Task 3 and the third part introduced the participants to cat’s eyes. Students were encouraged to speak their thoughts candidly.

##### Exploring SLDs

**FIGURE 7:**
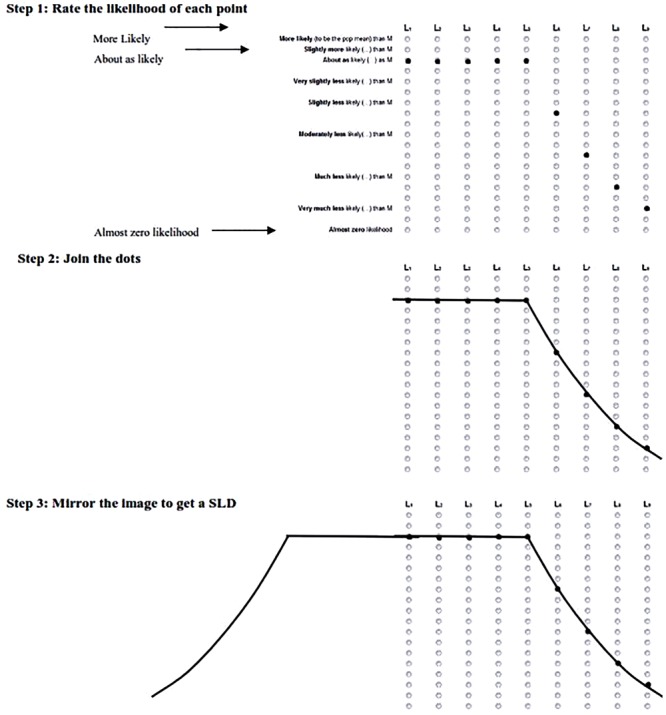
Three steps used to elicit students’ CIs during Section “Introduction” of the interview. 5 1: dots marked by interviewer, to represent that student’s responses to Experiments 1, 2. Student joins the dots, 3. Interviewer extends the shape across the page to mirror the shape and draw the students SLD. L_1_ to L_9_ represent the corresponding points on **Figure 2**.

Part 1 began by picturing the students’ SLD as elicited in Task 2 of Experiment 1. **Figure 7** shows the steps taken to guide the students to draw their SLD. First, students referred to **Figure 2** and were shown their responses from Task 2 to each point inside and outside a CI (L_1_ to L_9_) landing on the μ relative to the sample mean. The students then joined each of the dots by drawing on the scale. Finally, they were asked to mirror the image on the other side of the interval, thus completing a figure of their SLD. We gauged the students’ reaction to this shape, and if they decided to change the shape we asked about the previous shape (from Experiment 1), the new shape, and why their intuitions changed.

##### Exploring the relationship between interval length and C% level (Task 4)

The second task discussed in the interview focused on students’ intuitions about *C*% level and CI length. During the interview we asked the students about the effects of increasing and decreasing *C*% level on the length of a CI.

##### Exploring the effect of cat’s eyes

Part 3 of the interview introduced students to the novel concept of cat’s eyes. First, the students saw a CI and the on-screen controls were explained these controls enabled the students to change N, SD, toggle the overall distribution and the cat’s eye (the amount of shaded area inside the overall distribution represented by the interval) on and off. Then the task was explained; the interviewer would ask the student to make a series of guesses about changes in length given *C*% level and *N* before asking them to explore the program. The student would then be asked to try to explain what was happening on screen as they experimented with changes in *N, C*% level and CI length. The students reflected on their previous answers in light of this new information.

##### Data transcription and coding

The audio from student interviews was recorded then independently transcribed by a research assistant (*N* = 5) or the first author (*N =* 19). The research assistant’s transcriptions were then checked by cross referencing them to the audio recordings and no corrections were needed.

Coding sheets and a coding manual were developed and each interview was independently double coded. The coding process involved identifying each mention of 22 possible concepts. When a misconception or correct conception (from the list 1.1 to 4.5 in Results below) was found in a students’ interview transcript it was coded as *present.* If in the interview students explicitly stated that they did not believe a concept (misconception or correct conception) the code *explicit absent* was applied. If a student simply did not mention a concept it was coded *implicit absent.* Finally, if a student spoke around a concept without quite identifying it, or expressed explicit confusion over concept, the code used was *unsure.* The mean inter-rater reliability was Cohen’s Kappa = 0.81 with a range of 0.74 to 0.90 for the 22 concepts which is acceptable given the complexity of the coding task.

### Results

Twenty-one of the concepts (both CI misconceptions and correct CI conceptions) investigated were developed *a priori* and largely informed by previous statistical cognition research in our laboratory, specifically Cumming et al. (2004), Fidler and Loftus (2009), and Experiment 1. After coding all 24 interviews one misconception “as *N* increases *C*% level increases” was added *post hoc*.

Overall every participant held at least one CI misconception, with a mean of 4.6 misconceptions per participant. At the end of the interviews (after exposure to cat’s eye CIs) the mean number of misconceptions dropped to 2.0 and one participant had no CI misconceptions.

The conceptions were grouped into four categories. Three categories were misconceptions: definitional, relational, and shape. The fourth category comprised correct conceptions.

#### Definitional Misconceptions

We coded six ‘Definitional’ misconceptions, which reflect misunderstandings about what a CI is or represents. The following is a list of definitional misconceptions, followed by an example quote from the interviews.

*1.1 The Confidence Level Misconception* (Cumming et al., 2004).

“You can be 95% confident that if you replicated the study the mean will fall within this gray area.” (Student #21)

*1.2 A CI is a range of plausible values for the sample mean* (Fidler, 2005, unpublished).

[Interviewer] “What does the [Confidence Interval] represent?”
[Student] “That’s where the sample means can be considered to be within the 95% CI” (#9)

*1.3 A CI is a range of individual scores* (Fidler, 2005, unpublished).

“The [*C*% level] refers to the amount of SDs from the mean and how much of the data falls within those points.” (#8)

1.4 A Standard error (SE) is the same as a standard deviation (SD) (New).

This misconception could either be due to simple miscommunication or a genuine misunderstanding about the difference between SDs and SEs. Of course any SE is actually a SD—of a sampling distribution. But we are making the extra assumption that by SD a student means the SD of the data, not of the sampling distribution of the sample mean.

“I remember I know that a CI [is] kind of four SDs wide.” (#4)

1.5 A 50% CI indicates lack of data (New).

“I’m not quite sure but I suppose having a 50% CI means you probably don’t have a lot of data… with a 95% CI you probably have a wider range of data and therefore can make a larger percentage judgment of where the μ might lie.” (#5)

1.6. A 50% CI means the μ could land anywhere (New).

“[A 50% CI means] It means it’s just as equally likely as it is unlikely… so if I were to like flip a coin the mean might be there, if I did it again it might be there and if I did it again it might be down there because it’s 50–50. And it …if it’s 50% in here and 50% outside, it means it’s the same [likelihood] all across the board.” (#14)

#### Relational Misconceptions

‘Relational’ misconceptions are misconceptions about how the different aspects of a CI relate, for example, how length changes as a function of *C*% level and *N*.

2.1 As N increases, length does not change (New).

“It [CI length] stays the same [as sample size increases], it just stretches up and down… it wouldn’t do that in real life as there is no point in doing that but it stays the same.” (#14)

2.2 As N increases, length increases, and as N decreases, length decreases (New).

“If you have more participants… for some reason my intuition [is that the CI] just gets wider because you cover more range.” (#24)

*2.3 As C% level increases, length decreases* (Fidler, 2005, unpublished).

“I guess you’re casting the net wider [as *C*% level decreases] … Yeah, because we are less sure [the net gets wider]. The more confident we are, the smaller the net we need…because we kind of know where it’s going to fall.” (#12)

*2.4 Overlap misconception* (Belia et al., 2005).

Interviewer: “with independent groups, you are looking for an overlap of less than a quarter of the CIs to be statistically significant at 0.05.
Student: “I thought they couldn’t overlap at all?”

2.5 As N decreases C% level decreases as N increases C% level increases, (Added post hoc, New).

“So if you reduce the sample size the CI should go wider because you are less confident.” (#24)

#### Shape Misconceptions

Shape misconceptions are misconceptions about the shape of the distribution underlying a CI. One of these CI shape misconceptions (the cliff effect) was previously identified by Lai et al. (2012).

3.1 Everything inside the CI is equally likely.

“My reasoning was CI just tells you that I’m 95% confident that the mean falls within this range but it doesn’t mean that [if a point] is closer to the mean, it doesn’t tell you that it’s more likely to happen.” (#24)

3.2 Everything outside the CI is equally unlikely.

“The others [points inside the CI] are equally likely and these [points outside the CI] are equally unlikely.” (#22)

3.3 Cliff effect.

“Well that point is just outside the CI so it’s much less likely.” (#15)

3.4 Standard shape regardless of C% level

“So the likelihood that the mean decreases is the same regardless of the percentage of the CI.” (#13)

3.5 Linear reduction in likelihood across a CI.

“The further away you move from the mean the less likely it is to be representative of the μ, and [I was] really just working on predominantly a linear scale but I kind of ran out of room, perhaps if I could do it again I’d move it all backward so it would look a bit more linear.” (#23)

3.6 A 95% CI is double the length of a 50% CI.

“[A 95% CI is] just under double [the length of a 50% CI]. One full 50% CI would fit in one of the MOEs (margin of error; i.e., one arm) of the 95%CI.” (#21)

#### Correct Conceptions

We were also interested in students’ correct conceptions. ‘Correct conceptions’ are those which fit with normative statistical theory.

4.1 C% level indicates area under the curve.

“We are looking at a smaller area of the distribution.” (#17)

4.2 There is a normal distribution.

“I was trying to represent the curved nature… how the normal distribution curved and tapered off.” (#4)

4.3 A 50% CI is a portion of a 95% CI.

“So when we start with a 50% CI a 95% CI would have to cover a lot more area than twice the amount. Probably two and a half times the amount.” (#1)

4.4 Increasing N decreases CI length.

“Well assuming that the results stay similar [the length] would decrease, as long as you are not introducing more variability to the data… say the extra data is something totally different from the first data.” (#5)

4.5 Gradual decrease from the sample mean.

“… it just decreases in likelihood as we get away from the mean.” (#10)

#### Base Rates and the Effect of Cat’s Eyes

**FIGURE 8:**
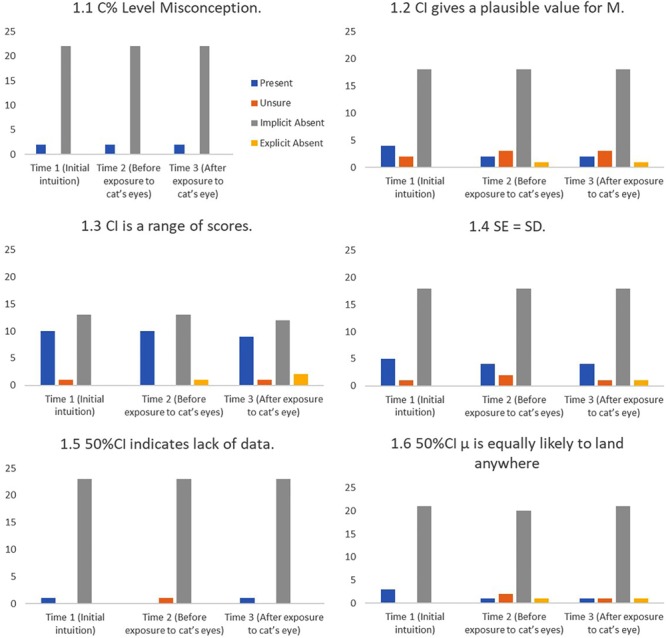
Frequency of students stating intuitions at first mention (Time 1), at last mention immediately before exposure to cat’s eye program (Time 2), and after exposure to cat’s eye program (Time 3) for definitional misconceptions (*n* = 24).

**FIGURE 9:**
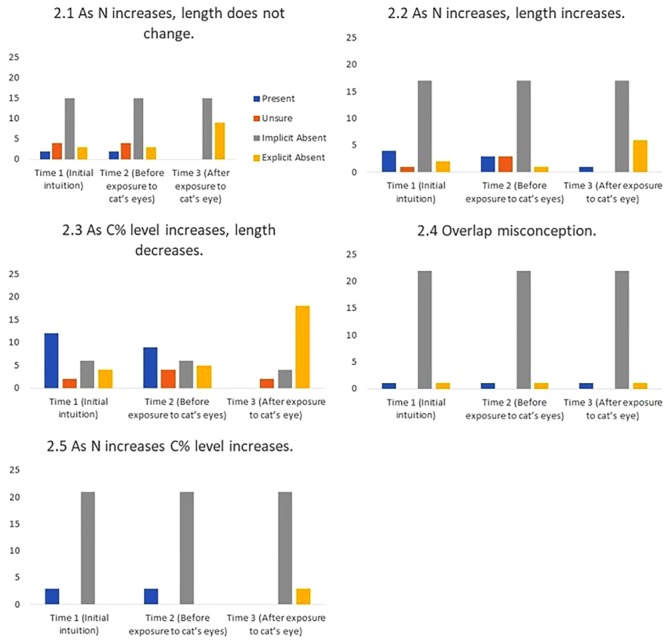
Frequency of students stating intuitions at first mention (Time 1), at last mention immediately before exposure to cat’s eye program (Time 2), and after exposure to cat’s eye program (Time 3) for relational misconceptions (*n* = 24).

**FIGURE 10:**
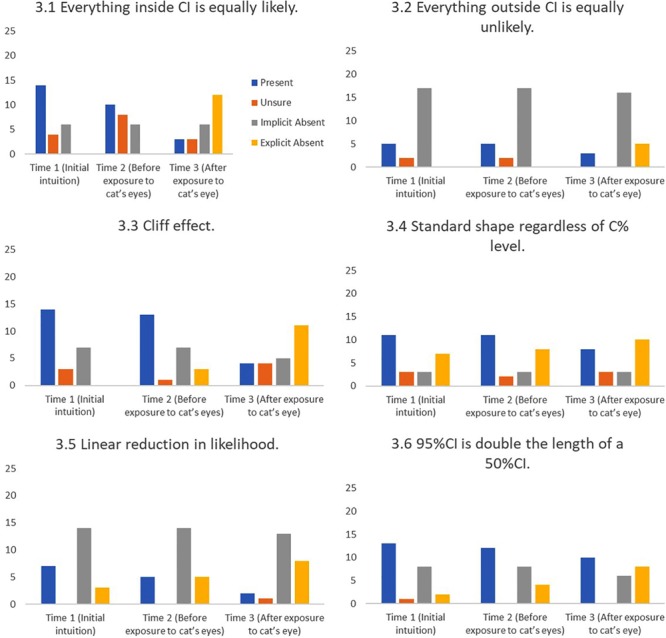
Frequency of students stating intuitions at first mention (Time 1), at last mention immediately before exposure to cat’s eye program (Time 2), and after exposure to cat’s eye program (Time 3) for shape misconceptions (*n* = 24).

**FIGURE 11:**
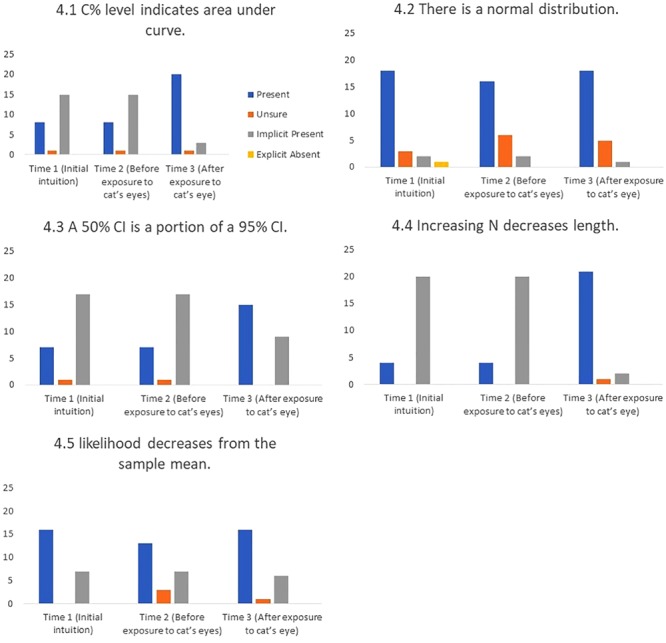
Frequency of students stating intuitions at first mention (Time 1), at last mention immediately before exposure to cat’s eye program (Time 2), and after exposure to cat’s eye program (Time 3) for correct conceptions (*n* = 24).

**Figures 8–11** show the presence of each of the 22 misconceptions and correct conceptions at three time points during the interview. Time 1 and Time 2 occur before exposure to cat’s eyes. Time 1 is the first mention of the concept and therefore the baseline for the misconceptions in this sample. Time 2 is the last mention of the concept before exposure to cat’s eyes. Time 3 is after exposure to cat’s eyes and is the final intuition before the end of the interview. Note that if a misconception is only mentioned once before the exposure to cat’s eyes, Times 1 and 2 are the same, and represent the same statement.

*Cat’s eyes and definitional misconceptions.* **Figure 8** shows that introducing cat’s eyes to participants did not change the definitional misconceptions of many participants.

*Cat’s eyes and relational misconceptions.* **Figure 9** shows that, in stark contrast to definitional misconceptions, cat’s eyes were effective at reducing relational misconceptions.

*Cat’s eyes and shape misconceptions.* **Figure 10** suggest that cat’s eyes were effective at removing misconceptions about the shape of a CI’s underlying distribution.

*Cat’s eyes and correct conceptions.* **Figure 11** shows that cat’s eyes were effective at encouraging students to express correct conceptions about CIs.

To check whether introducing the cat’s eye had a effect on students’ intuitions we ran a Wilcoxon Rank Sign test for each intuition. The rankings of the responses were coded as: For misconceptions (Categories 1–3) the following rankings were used Present = 0, Unsure = 1, Implicit Absent = 2, Explicit Absent = 3.

For the correct conceptions the rankings were Present = 3, Implicit Present = 2, Unsure = 1, Explicit Absent = 0. **Table 4** shows the *Z*-scores and *p*-values for the Wilcox Rank Sign test.

**Table 4:** Wilcox Rank Sign Test for student misconceptions at baseline (Time 1), at last mention immediately before exposure to cat’s eye program (Time 2), and after exposure to cat’s eye program (Time 3).

	Time 1 to Time 2		Time 2 to Time 3	
Intuition	*Z*	*p*	*Z*	*p*
1.1	–1	0.317	1	0.317
1.2	–2.07	0.038	0	1
1.3	–1.41	0.157	–2.165	0.03
1.4	–1	0.317	–1.414	0.157
1.5	–1	0.317	–1	0.317
2.1	0	1	–3.272	0.001
2.2	–1	0.317	–2.06	0.039
2.3	0	1	–2.549	0.011
2.4	0	1	–2.232	0.026
2.5	0	1	–3.491	<0.001
3.1	–1.089	0.276	–3.109	0.002
3.2	–0.772	0.47	–1.823	0.068
3.3	–1.414	0.157	–1.89	0.59
3.4	–1.342	0.18	–1.89	0.059
4.1	0	1	–3.464	0.001
4.2	0	1	–1	0.317
4.3	0	1	–1.414	0.157
4.4	0	1	–1.493	0.135
4.5	–1.414	0.157	–1.134	0.257

### Discussion

By interviewing students we confirmed that the misconceptions discussed in Experiment 1 were held by some students and that the measures we used to elicit SLDs had good construct validity. In Hoekstra et al. (2014) and Garcia-Perez and Alcala-Quintana (2016) studies, students and researchers were asked to endorse from a number of presented statements. Conclusions from this research (although informative) is limited as results may merely indicate the participants’ willingness to endorse statements rather than reflect participants true intuitions about CIs. This experiment allowed students to express their own interpretations of CIs.

Student quotes demonstrated both misconceptions and correct conceptions that students have about CIs. In addition, interviews identified a misconception not previously described in the literature, that the sample size has an effect of the *C*% level.

Initially we had expected to find clusters of definitional and relational misconceptions that could link to false beliefs about CI distributions. In reality, students’ understanding is too fragile and our sample size too small for any robust patterns to be identified. Instead the frequently diverse responses to the different tasks suggest that students often hold several competing intuitions (good and bad), and that misconceptions about shape do not translate into logically consistent relational misconceptions. Overall, cat’s eyes reduced the number of definitional misconceptions by 25%, relational misconceptions by 91%, and shape misconceptions by 53%.

As expected, there were few mentions of definitional misconceptions. Also, exposure to cat’s eyes had no substantial effect on removing the definitional misconceptions mentioned. This can be explained by the focus of the interview. Students were never asked to define a CI. Instead students were asked to clarify any definitions they gave. Also the cat’s eye program was designed to improve students’ SLDs and reasoning on the effects of sample size *C*% level on the length. The program was not designed to explain what the CI is estimating or how a CI is calculated.

The interviews were much more successful at eliciting relational and shape misconceptions. Cat’s eyes were effective in removing these misconceptions in most but not all cases. Some students still found the relational and shape concepts challenging even when provided with a tool that demonstrates relational and shape concepts. One student reflected on why a CI is such a difficult concept:

“I guess it’s hard just because there are so many things to hold in your head at the same time. There is the distribution, there is the *C*% level, there is the CI and its [length]… you’re trying to map all these things on top of each other and get a shape.” (#14)

The cat’s eye makes explicit the normal distribution underlying the CI (Cumming, 2007, 2012). The area of the distribution is constant and the height and length of the interval are inversely proportionate as *N* and the *SD* vary. By changing *N* or the *SD* the overall distribution gets longer and thinner or shorter and fatter (taller). The amount of area shaded reflects the *C*% level and so the relationship between *C*% level and area is also explicit. Because the display is interactive, students can explore this relationship. After exploring the cat’s eye program, Student #14 explained how her concepts of these relationships changed:

“I don’t think I could reason that [the interaction between length, *C*% level and *N*] in my head. I’d actually have to see it. When you asked about increasing the sample size, I was thinking you’re going to narrow [the interval] in, on your sample mean, you’re going to be more confident that [μ is] there. But then the CI itself gets smaller so what does that mean? The CI was getting longer and shorter because of the *C*% level before. So I couldn’t rectify those ideas. But then when I saw it visually it made sense. I didn’t think about the distribution itself coming in. So it’s not like the *C*% level is changing, it’s the distribution that’s changing and its bringing [the *C*% level] in with it. There is nothing really changing it’s just squishing.” (#14)

The cat’s eye was particularly helpful at reducing the relational misconception about *C*% level and length. Approximately one third (42%, 10 of 24) of the interviewed students initially believed that a 95% CI would be shorter than a 50% CI, given the same data. After seeing the cat’s eyes, students could identify this as a misconception. Here is a statement of Student #11’s initial belief:

“The larger the CI, the less confident we are of the [results]. The smaller the CI, the more power a study would have, the more confident we are of the results.” (#11)

Here is the same student gaining insight through the cat’s eye:

“So changing the *C*% level is changing the amount of the distribution that you are looking at. So 95 is 95% of the distribution and 50 is 50%…so hang on… so it gets shorter.” (#11)

Misconception 2.2; ‘length of a CI increases as *C*% level decreases’ was surprising. We did not expect many postgraduate students to make this error. When explaining, students often equated confidence with precision:

“A 95% CI is smaller because you are more certain that the μ lies within this area. The 50% you are less certain as to where the population mean is so you have to widen your… scope.” (#5)

This idea of equating confidence to precision is also present in our previous quote by student (#12). As mentioned, in colloquial language, a highly confident person gives a precise estimate while a person with low confidence in their guess will often give a vaguer estimate. This intuition would logically also be responsible for the misconception ‘a 50% CI means we lack data.’ A person with little information would not be very confident about any guess they made. In fact student (#5) had both of these misconceptions.

Cat’s eyes also helped students understand that the underlying distribution of a CI was a normal distribution. Fourteen (of 24) participants interviewed mentioned that they thought that a CI had an underlying uniform distribution, meaning all points inside the CI were equally likely to land on the μ:

“Basically if you can say 95% of the time [the μ] is within the CI, then that means that any of these points are equally likely along that line.” (#18)

Exposure to the cat’s eyes removed this misconception for all but three of these participants. For example the student quoted above responded:

“The confidence interval, it represents… the curve represents the likelihood that a point is the μ.” (#18)

However, not all participants were satisfied by this:

“If [μ] is there (M), we don’t know that it’s definitely there, or definitely there (points to upper limit). So you can’t say that something is less likely. It could fall anywhere in that range… I suppose I could say it’s less and less likely because that’s what the picture tells me, but it doesn’t tell me why.” (#22)

#### Conflicting Intuitions

One interesting insight gained from these interviews is that although the tasks we presented to the students were understood and were valid as representations of the students’ intuitions, students’ intuitions were fuzzy, conflicting but coherent. Students were able to communicate their intuitions and even reason them out despite the intuitions sometimes conflicting with one another. As mentioned, the relationship between *C*% level, sample size, and length was difficult to conceptualize for several students. Given that both *C*% and sample size affect length sometimes the students became confused. For example

Interviewer (I): “What would happen to a CI when we decrease the *C*% level, say from 95 to 50?”
Student (S) (#17): “I would say it gets smaller”
I: “What if we decreased the number of participants?”
S: “It would get bigger… because wouldn’t you be less certain?”
I: “Let’s go with what you are thinking.”
S: “Well if there are less participants then you are less certain so that means you have to make the CI itself smaller, like a smaller percentage …and then if you did that I would…Oh no! Now everything is conflicting! If it is smaller, then doesn’t that mean it’s better? Or…No wait…I don’t know!

Student #17 has basically correct intuitions when each concept is presented separately. When the concepts are presented together the student was conflicted. She understood that decreasing the sample size increases the uncertainty (by reducing the number of participants, and therefore increasing variability). She also believed that decreasing the *C*% level reduces the length of the CI, and that researchers prefer precise CIs. All of her intuitions were correct. Unfortunately she also linked an increase in variability with a decrease in *C*% level. When answering these questions separately, each of these intuitions were unchallenged. The conflict only became apparent when the student was asked to explain her intuitions.

#### Unexpected Directions

Interviews also provided some new unexpected directions for future research. Student #13 provided a SLD that indicated the likelihood of capturing μ initially increased as it moved away from the sample mean before gradually dropping off.

Initially we thought that the student made a mistake and was trying to plot a normal distribution. The interview provided a much more interesting explanation for this SLD:

Interviewer (I): “One thing I noticed is that [point L_2_ and L_3_] are more likely to be the μ than the sample mean, did you mean that?”
Student #13 (S): “Yes”
I: “If you don’t mind let’s go behind your reasoning…”
S: “I always overestimate.”
I: “What did you mean by ‘you always overestimate’?”
S: “I just [have to] account for variables I haven’t thought of before.”
I: “So when you don’t account for variables, what does that mean? Does it mean you’re likely to get the estimate wrong or that you don’t have faith in your sample mean?”
S: “I’m just not 100% that it’s the correct mean, that it’s most likely reflecting the μ.”
I: “How is it [the μ] more likely to be around the mean but not the sample mean?”
S: “Um…I don’t know. I just don’t take the [sample] mean as a correct reflection so I always go outside the [sample] mean.”

Student #13 seems to have correct conceptions about sampling variability. She is correct that the μ is much more likely to land within the interval between L_1_ and L_3_ than to land on exactly the sample mean. She also seems to understand why error bars are so important. There may well be variables that a researcher has not considered that may make the sample mean an incorrect estimation. These correct intuitions ignore two normative principles: First, that any interval will take up more area of a likelihood distribution compared with any single point. Second, that researchers often make the assumption of a normal distribution when running statistical tests to account for variability in sampling data (but perhaps not other variables that have not been considered) using this assumption. Overall Student #13 showed correct conceptions about variability but did not seem to understand how variability is represented using CIs.

#### Limitations

One limitation of the study was participant fatigue. After a 45-min interview about statistics several participants seemed exhausted and may not have had motivation or energy to address their misconceptions with care. Also, any improvements found in the interviews after introducing cat’s eyes may have been merely temporary and not a stable and permanent conceptual change. Long term follow ups were beyond the scope of this study. A classroom intervention with follow ups could measure this using a shorter more direct qualitative survey to reduce participant fatigue Nonetheless, for many (71%) participants in our study, cat’s eyes helped with some difficult relational concepts. We also did not have a control or comparison intervention—conceivably there may be a simpler intervention that may improve intuitions.

To reduce investigator bias, a standard interviewing script was created and piloted several times before the first participant was interviewed. The script was identical for all participants. However, the interviewer and the participants were able to depart from the script when necessary and it is possible that on occasion some implicit investigator bias may have colored the participants’ responses. To further reduce investigator bias in the coding process all interviews were independently coded by a research assistant with an adequate mean Cohen’s Kappa of 0.81.

One methodological limitation involved the phrasing of the text presented to describe the 19 points in **Figure 7**. The participants were asked to rate the points relative to the mean. In the text all points are comparative, however, the final point is absolute. This means that a student could agree with the final statement “almost zero likelihood” as well as any other point simultaneously. Fortunately, there is no evidence that students did not interpret the instructions as intended. If a student was confused by the instructions the interviewer was present to further explain the task as intended.

Finally, our sample was small, and a sample of convenience. The proportion of students holding any conception does not necessarily provide a good estimate of the proportion holding that conception in the population of all graduate students. Experiment 1 gives a better estimate of the proportions of students with relational and shape misconceptions. For example, in our previous survey, 25% of students believed that as you increase a *C*% level of a CI the length decreases; in the second it was 42% of students.

### Conclusion

Overall the educational implications of using cat’s eyes are promising. We argue that they are a useful conceptual tool for students to encounter and discuss and maybe useful to keep in mind as a guide for thinking about and interpreting CIs. From this study a very minimal intervention has produced reasonably favorable results, at least in the short term. By providing a computer simulation that helped students explore and experience the relationships between length, *C*% level, sample size, and shape using cat’s eyes, we was able to observe and explore how students’ intuitions change and reason out why their intuitions were mistaken in the first place.

## General Implications

Interviewing participants in Study 2 triangulated the evidence from Experiment 1. Students do have varying SLD shapes and these shapes reflect how they think about CIs. Also, the measures used to plot students’ SLDs have good construct validity. In addition, the interviews confirmed previously reported CI misconceptions and have revealed new CI misconceptions. Students were able to articulate their intuitions and in some cases justify them. The interview data suggest that cat’s eyes improve student intuitions, particularly misconceptions about likelihood distributions, and relational misconceptions. Students seem to benefit from exploring their intuitions and testing whether these intuitions match with the cat’s eye program. Finally another important implication from the results is that students are able to hold several seemingly contradictory intuitions at one time, such as the presence of a normal distribution and the idea that everything inside the CI is equally likely to land on μ.

## General Conclusion

Studies 1 and 2 have provided a narrative on common misconceptions as well additional insights into how students think about CIs. It’s reasonable to assume that many researchers and clinicians also hold such misconceptions about CIs (although such claims would need to be verified). The much advocated position of moving to estimation (effect sizes and confidence intervals) is unlikely to return substantial benefits if CIs are routinely misinterpreted, or merely used as a substitute dichotomous decision making criteria. It is important to provide students, researchers and clinicians with easy to use, and intuitive tools that can help them overcome CI misconceptions. Cat’s eyes are promising to be quite effective at improving SLDs and reducing relational and shape misconceptions.

## Ethics Statement

This study was carried out in accordance with the recommendations of Human Research Ethics Guidelines, University Human Ethics Committee’ with written informed consent from all subjects. All subjects gave written informed consent in accordance with the Declaration of Helsinki. The protocol was approved by the University Human Ethics Committee.

All research reported in the paper were approved by the La Trobe Human Ethics Committee: HEC Approval Number 06-171.

## Author Contributions

PK designed the study and analyzed the data. PK also wrote up the initial draft. JL was involved with the design of the study and the drafting of the paper. GC was the principal supervisor overseeing the study, guiding the analysis and interpretation of the results. GC also was involved with drafting the paper.

## Conflict of Interest Statement

The authors declare that the research was conducted in the absence of any commercial or financial relationships that could be construed as a potential conflict of interest.

## Footnotes

1 http://www.psychologicalscience.org/index.php/publications/journals/psychological_science/ps-submissions
